# Layer-by-layer assembled 2D nanocomposites for extreme polarization optics

**DOI:** 10.1093/nsr/nwae353

**Published:** 2024-10-09

**Authors:** Jiao Yang, Qunfeng Cheng

**Affiliations:** School of Chemistry and Materials Science, University of Science and Technology of China, China; Suzhou Institute for Advanced Research, University of Science and Technology of China, China; School of Chemistry and Materials Science, University of Science and Technology of China, China; Suzhou Institute for Advanced Research, University of Science and Technology of China, China; School of Chemistry, Key Laboratory of Bio-inspired Smart Interfacial Science and Technology of the Ministry of Education, Beihang University, China; Institute of Energy Materials Science (IEMS), University of Shanghai for Science and Technology, China

Two-dimensional nanoplatelets with notable electrical, thermal, optical and mechanical properties have been used in many applications [1–4], including energy storage, water purification and electromagnetic interference shielding. Except for the intrinsic properties of 2D nanoplatelets, stacking morphology also affects the performance of the resultant 2D nanocomposites. For instance, aligned and compact stacking structures can induce excellent mechanical properties [4,5]. Stable and adjustable interlayer spacing improves ion selectivity and water-purification efficiency [3]. Properly designed ion transport channels and wrinkles can enhance the energy density of 2D nanocomposites [6,7].

A landmark study recently published in *Nature* [8] by Richard A. Vaia, André Farias de Moura, Dhriti Nepal and Nicholas A. Kotov demonstrated a layer-by-layer (LBL) strategy for assembling nano-achiral and partially disordered 2D nanoplatelets into polarization-active nanocomposites, which can be used in extreme environments with the operating temperature as high as 250°C. The assembled 2D nanocomposites have an optical asymmetry g-factor of 1.0, which exceeds those of typical nanomaterials by ∼500 times.

**Figure 1.**
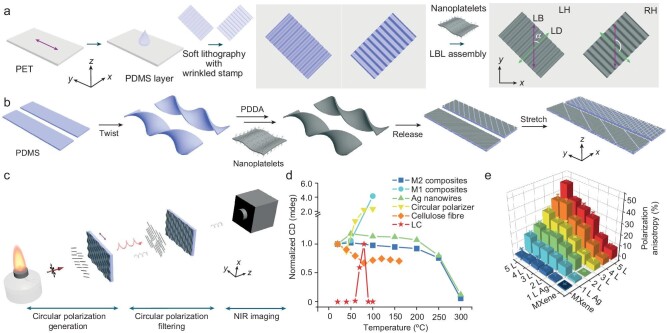
(a) Schematics of the preparation of polarization-active 2D nanocomposites by using soft lithography with wrinkled stamps. (b) Schematics of the preparation of polarization-active nanocomposites by using twist substrates. (c) Schematics of circularly polarized optical imaging with left-handed (LH) and right-handed (RH) nanocomposites. (d) Dependence of CD on temperature for different nanocomposites. (e) Dependence of the polarization anisotropy for flame with LH NIR polarization. Data are mean ± SD. Adapted with permission from Ref. [8].

As shown in Fig. 1a and b, Ti_3_C_2_T*_x_* nanoplatelets were used as an example. Ti_3_C_2_T*_x_* nanoplatelets were assembled on the substrates with wrinkled stamps by using the LBL strategy (Fig. 1a). The obtained 2D nanocomposites show linear birefringence (LB) and linear dichroism (LD). Also, the LB and LD could be achieved by LBL-assembling Ti_3_C_2_T*_x_* nanoplatelets on the twist substrates (Fig. 1b) because the 2D nanocomposites with cracked and wrinkled patterns on the compressed and stretched sides were obtained after a releasing process to flatten the twisted assemblies.

The excellent thermal stability of the Ti_3_C_2_T*_x_* nanoplatelets enabled the high thermal resilience of the 2D nanocomposites, so they could be used as circular modulators for polarized imaging of hot emitters, particularly in the near-infrared (NIR) range (Fig. 1c). They exhibit intense circular polarization even at 250°C, while commercial circular polarizers lose their optical activity at 50°C, cholesteric liquid crystals lose their optical activity at >90°C (Fig. 1d). Also, the optimized polarization contrast can be predicted through the sequence of LBL layers (Fig. 1e).

In short, Kotov and co-workers [8] provided a milestone study in the field of nanocomposites. The LBL strategy increased the simplicity of the manufacturing process. Materials modularity and computational predictability of the additively engineered nanocomposites enabled the assembly of the nano-achiral 2D nanoplatelets into polarization-active nanocomposites. A variety of optically active nanocomposites could be achieved, which will have a significant influence on extreme polarization optics.
